# Abstract of the Proceedings of the Buffalo Medical Association

**Published:** 1857-09

**Authors:** Sanford B. Hunt


ART. VI.—*Abstract of the Proceedings of the Buffalo Medical Association.*

TUESDAY EVENING, Aug. 4, 1857.

The association met.

Present—The Vice-President, Dr. Wyckoff, in the chair. Drs. Root, Nichols, Eastman, Fanner, Wilcox, Rogers, Rochester, Strong, Whitney, Ring, Newman, Hunt, Dayton, and Gay.

The minutes of the preceding meeting were read and approved.

Drs. Charles Fanner and Henry Nichell were elected to membership.

The committee on puerperal convulsions asked further time to report. Granted.

*Prof. Rochester* reported a case of rheumatism remarkable for the variety of organs it attacked and in proving fatal. The patient was a healthy and robust man, a soap and candle maker by trade, and of intemperate habits. When first attacked he was suffering from what appeared to be bilious cholic, but Prof. R. diagnosticated rheumatism of the muscular coat of the bowels. This was relieved when a second attack upon the diaphragm occurred followed by metastasis to the knee, then to the heart, a bellows murmur accompanying its sounds, then again to the diaphragm. The bellows murmur ceased after this metastasis. Receding from the diaphragm it involved the muscular coat of the stomach. The dyspnœa and pain in the midriff were replaced by general prostration, vomiting, and extreme irritability of the stomach. During all these changes about four weeks of time were consumed during which the patient steadily lost ground. From the stomach the disease progressed to the muscular coat of the bowels and then attacked the lung.

The symptoms were those of pneumonia, but not of the frank form. There was little cough and only once rusty sputa. There was, however, a distinct crepitant râle. From this he was relieved under treatment, but a noteworthy fact is that immediately on the subsidence of the pneumonia the rheumatism immediately renewed its onset on the bowels, an incident of disease mentioned by La Roche in his work as not very uncommon in rheumatic pneumonia.

The rheumatism of the bowels was followed by dysentery, and at the end of the eighth week he seemed free from everything except serious prostration. About the middle of July, he was left one afternoon in a comfortable and apparently convalescent condition, but Dr. Rochester found him next morning, suffering from meningitis, which was followed rapidly by effusion and death. Whether the meningitis was rheumatic or not Dr. Rochester was not fully satisfied. At times during the progress of the disease, the patient had had symptoms of delirium tremens, from which, however, he had been quite free for some time before his death.

Prof. Rochester had seen one other fatal case of rheumatism. It was at first lumbar, then underwent a metastasis to the knee, where it became persistent; abscess formed, followed by phlebitis and consequent death.

*Dr. Wyckoff* also related a fatal case of rheumatism. A gentleman, aged 34 years, whose habits had been for some months intemperate, was taken with mild lumbar rheumatism, which passed down to the hips and thence to the knees, which latter were very much swollen for four or five days, when it went to the ankles and thence to the toes. After this were attacked, successively, the elbow, shoulder and wrist of one arm, and next the other arm. Later in the disease, he suffered from bronchial trouble, followed by congestion first of the right and then of the left lung; but he finally seemed convalescent from this. Dr. Wyckoff left him one evening appearing comfortable. During the night he slept, at first, well, then became incoherent and delirious, but the nurse not being aware of his danger, delayed sending until morning. At 7, A. M., Dr. W. found him apoplectic, comatose, and with stertorous breathing. He died at 8½, A. M.

*Dr. Wilcox* alluded to a case which he had reported some time ago, with reference to the question whether a syphilitic taint existing in the system was not sometimes roused to activity by rheumatic attacks. The patient alluded to had suffered from rheumatism which was evidently connected with syphilis, but, when apparently improving, had serous apoplexy, and died.

An inquiry was made as to the prevalence of summer diseases. Very little of diarrhœa or its congeries had been seen.

And the association then adjourned.

SANFORD B. HUNT, Secretary.

